# Non-contiguous finished genome sequence and description of *Halopiger djelfamassiliensis* sp. nov.

**DOI:** 10.4056/sigs.4578289

**Published:** 2013-10-05

**Authors:** Ikram Imene Hassani, Catherine Robert, Caroline Michelle, Didier Raoult, Hocine Hacène, Christelle Desnues

**Affiliations:** 1 USTHB Université, Laboratoire de Biologie Cellulaire et Moléculaire, Faculté de Biologie Algérie; 2 Aix-Marseille Université, Faculté de médecine, France.

**Keywords:** *Halopiger djelfamassiliensis*, Draft genome, *Archaea*, Halophile

## Abstract

*Halopiger djelfamassiliensis* strain IIH2^T^ sp. nov. is the type strain of *Halopiger djelfamassiliensis* sp. nov., a new species within the genus *Halopiger*. This strain, whose genome is described here, was isolated from evaporitic sediment of the hypersaline Lake Zahrez Gharbi in the Djelfa region (Algeria). *H. Djelfamassiliensis* is a Gram-negative, polymorphic-shaped and strictly aerobic archaeon. Here we describe the features of this organism, together with the complete genome sequence and annotation. The 3,771,216 bp long genome-contains 3,761 protein-coding and 51 RNA genes, including 4 rRNA genes.

## Introduction

*Halopiger djelfamassiliensis* sp. nov. strain IIH2^T^ (= KC430939 = DSM on-going deposit) is the type strain of *H. djelfamassiliensis* sp. nov. It is a Gram-negative, aerobic, non-motile and polymorphic archaeon that was isolated from evaporitic sediment of the hypersaline Lake Zahrez Gharbi in the Djelfa region (Algeria) as part of a project studying archaeal diversity in hypersaline Lakes of Algeria.

Classically, the classification of prokaryotes is based on a combination of phenotypic and genotypic characteristics [1] also known as polyphasic taxonomy. To date, only 192 archaeal genomes have been sequenced [2]. As the cost of genomic sequencing is constantly decreasing, the number of archaeal sequenced genomes is expected to grow in the next few years. We propose to describe new archaeal species by adding genomic information [3,4] to phenotypic criteria, including the proteic profile [5,6], as it was previously used for the description of new bacterial species [7-19].

The genus *Halopiger* created in 2007 by Gutiérrez [20], contains only three species, *Halopiger xanaduensis* SH-6^T^ isolated from the Shangmatala salt lake, Inner Mongolia, china [20], *Halopiger aswanensis* 56^T^ isolated from the surface of hypersaline salt soils close to Aswan, Egypt [21] and *Halopiger salifodinae* KCY07-B2^T^ recently isolated from a salt mine in Kuche county, Xinjiang province, China [22]. So far, this genus is composed of aerobic, Gram-negative, polymorphic and pigmented strains [20-22].

Here, we present a summary classification and a set of features for *H. Djelfamassiliensis* sp. nov. strain IIH2^T^ (= KC430939 = DSM ongoing deposit) together with the description of the complete genome sequencing and annotation. These characteristics support the circumscription of the *H. Djelfamassiliensis* species.

## Classification and features

**Figure 1:**
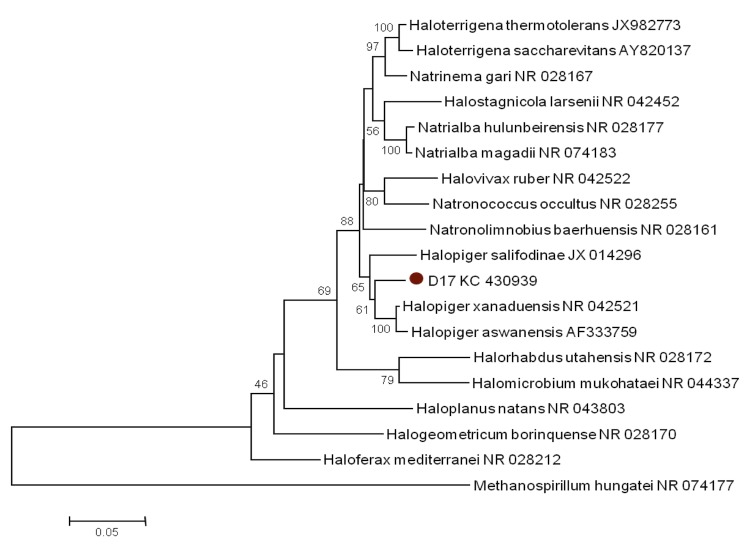
Neighbor-joining phylogenetic tree based on 16S rRNA gene sequence comparisons, showing the position of strain IIH2^T^ and some other related haloarchaeal species. GenBank accession numbers are indicated in parentheses. Sequences were aligned using MUSCLE, and phylogenetic inferences obtained using the MEGA software. Numbers at the nodes are bootstrap values obtained by repeating 1,000 times the analysis to generate a majority consensus tree. *Methanospirillum hungatei* was used as outgroup.

*Halopiger djelfamassiliensis* sp. nov. strain IIH2^T^ was isolated from evaporitic sediment of the hypersaline Lake Zahrez Gharbi in the Djelfa region of Algeria. Sediment samples (1g) were added to a 250 mL Erlenmeyer flasks containing 100 mL of SG medium [23] supplemented with ampicillin (100 μg/mL). Liquid enrichment cultures were incubated on a rotary shaking platform at 150 rpm for 7 to 10 days. After 1/10 dilution, aliquots (100 µL) were plated in SG medium supplemented with sterilized sediment extracts and incubated at 40°C for 7-30 days. In order to obtain pure culture, colonies were transferred to fresh solid SG medium. Strain IIH2^T^ (Table 1) was isolated in 2012 by cultivation in aerobic condition at 40°C. The strain exhibited a nucleotide sequence similarity with other members of the genus *Halopiger* ranging from 95% with *H. salifodinae* strain KCY07-B2^T^ to 96% with *H. xanaduensis* strain SH-6^T^ and *H. aswanensis* strain 56^T^, its closest validated phylogenetic neighbor (Figure 1). These values were lower than the 98.7% 16S rRNA gene sequence threshold recommended by Stackebrandt and Ebers to delineate a new species without carrying out DNA-DNA hybridization [32].

**Table 1:** Classification and general features of *Halopiger djelfamassiliensis* according to the MIGS recommendations [24].

MIGS ID	Property	Term	Evidence code ^a^
		Domain *Archaea*	TAS [25]
		Phylum *Euryarchaeota*	TAS [26]
		Class *Halobacteria*	TAS [27,28]
		Family *Halobacteriaceae*	TAS [29,30]
		Genus *Halopiger*	TAS [20]
		Species *Halopiger djelfamassiliensis*	TAS [31]
		Type strain IIH2^T^	IDA
	Gram stain	Negative	IDA
	Cell shape	Amorphous	IDA
	Motility	Non motile	IDA
	Sporulation	None	IDA
	Temperature range	Between 37°C and 55°C	IDA
	Optimum temperature	40°C	IDA
MIGS-6.3	Salinity	Halophile, 25% (optimum)	IDA
MIGS-22	Oxygen requirement	Aerobic	IDA
	Carbon source	Sugar or amino acids	IDA
	Energy metabolism	Heterotrophic	IDA
MIGS-6	Habitat	Salt Lake sediment	IDA
MIGS-15	Biotopic relationship	Free living	IDA
MIGS-14	Pathogenicity	Non-pathogenic	NAS
	Biosafety	1	NAS
	Isolation	Sediment of Zahrez Gharbi Lake	NAS
MIIGS-4	Geographic location	Algeria	IDA
MIGS-5	Isolation time	2012	IDA
MIGS-4.1	Latitude	34.916667	IDA
MIGS-4.2	Longitude	2.833333	IDA
MIGS-4.3	Depth	Surface	IDA
MIGS-4.4	Altitude	826 m	IDA

a) Evidence codes – IDA: Inferred from Direct Assay; TAS: Traceable Author Statement (i.e., a direct report exists in the literature); NAS: Non-traceable Author Statement (i.e., not directly observed for the living, isolated sample, but based on a generally accepted property for the species, or anecdotal evidence). These evidence codes are from the Gene Ontology project [31]. If the evidence is IDA, then the property was directly observed for a live isolate by one of the authors or an expert mentioned in the acknowledgements.

Phenotypic tests of strain were performed according to the proposed minimal standards for the description of new taxa in the order *Halobacteriales* [33]. Different growth temperatures (30, 37, 40, 50, 55, 60°C), pH (5, 6, 7, 7.5, 8, 8.5, 9, 10, 11, 12) and NaCl concentration (0, 10, 12, 15, 20, 22.5, 25, 30%) were tested. The requirement of Mg^2+^ for growth was determined in media containing 0, 1, 2.5, and 5g MgSO_4._ Growth occurred between 37°C and 55°C (optimum at 40°C), between 15% and 30% NaCl (optimum at 25% NaCl) and between pH 7-11 (optimum at pH 8). Mg^2+^ was not required for growth.

**Figure 2:**
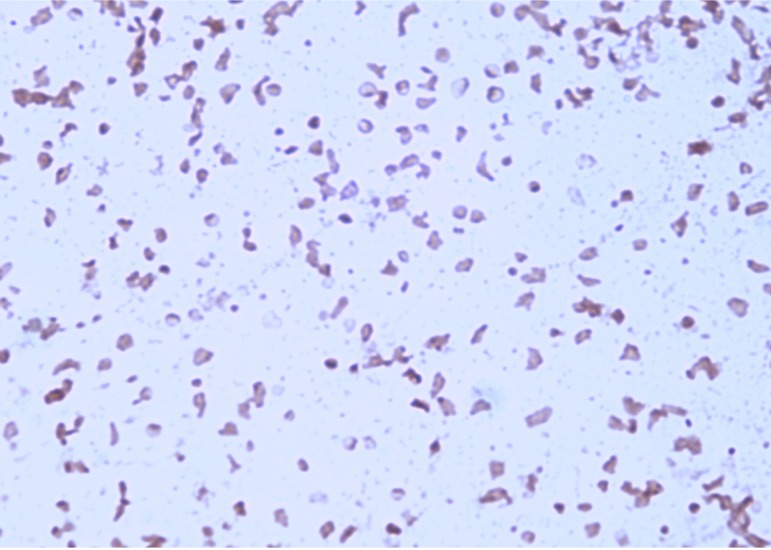
Gram staining of *Halopiger djelfamassiliensis* strain IIH2^T^.

**Figure 3:**
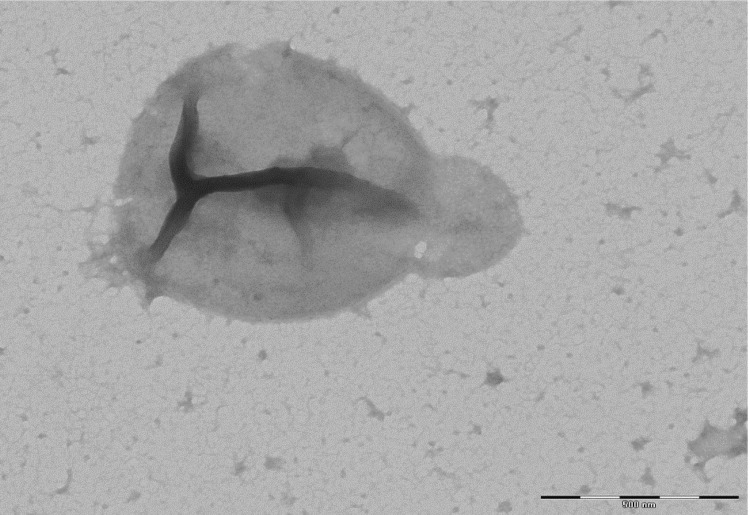
Transmission electron microscopy of *H. djelfamassiliensis* strain IIH2^T^, using a Morgani 268D (Philips) at an operating voltage of 60kV. The scale bar represents 500 nm.

Colony morphology was observed under optimal growth conditions on agar medium after incubation in aerobic conditions at 40° C for 7 days. The colonies of strain IIH2^T^ were cream-pigmented, viscous and smooth with a diameter of 3 to 4 mm. A negative result was observed in the motility test. Gram staining was performed following the method outlined by Dussault in 1955 [34]. Cells grown on SG medium agar were Gram-negative (Figure 2) polymorphic-shaped with a diameter ranging between 0.9 and 2.2 µm (Figure 3).

All the following biochemical and nutritional tests were realized in duplicate. Strain IIH2^T^ was found to be oxidase- and catalase- positive. Negative results were obtained for tryptophanase, β-galactosidase, arginine decarboxylase, H_2_S and indole production. Tween 80, gelatin, casein and lipids from egg yolk were hydrolysed at 40°C and 55°C, whereas urea, starch, and phosphatase were not. Methyl red and Voges-Proskauer tests were negative.

To estimate the utilization of various carbohydrates as carbon and energy sources, a minimum medium [250 g l^-1^ NaCl, 20 g l^-1^ MgSO_4_.7H_2_O, 2 g l^-1^ KCl, 0.1 g l^-1^ yeast extract (Difco), 0.5 g l^-1^ NH_4_Cl, 0.05 g l^-1^ KH_2_PO_4_, at pH 8.0] was supplemented with 1% of test carbohydrates. Strain IIH2^T^ can use as sole source of carbon and energy, organic nitrogen compounds such as casamino acids, peptone, tryptone and non-nitrogenous compounds such as acetate and pyruvate. Production of acids from carbohydrates was tested in the minimun medium supplemented with 0.5 g test substrate l^-1^. Phenol red was used as an indicator to detect acid production. Positive reactions were observed for D-glucose, D-melibiose, L-rhamnose, D-xylose, D-galactose, D-mannose, D-ribose and D-sucrose fermentation. No fermentation was observed with starch, fructose, D-lactose, dextran and mannitol. Table 2 summarizes the differential phenotypic characteristics of *H. djelfamassiliensis* sp. nov. IIH2^T^, *H. xanaduensis* SH-6T, *H. aswanensis* 56^T^ and *H. salifodinae* KCY076B2^T^.

**Table 2:** Differential phenotypic characteristics between strain IIH2^T^ and related species

**Characteristic**	***H. djelfamassiliensis***	***H.*** ***xanaduensis***	***H. aswanensis***	***H. salifodinae***
Cell morphology	pleomorphic	pleomorphic	pleomorphic	pleomorphic rods
Cell diameter (µm)	0.9-2.2	0.5-1.0×3.0-13.0	1.25-6.50×0.6–0.9	ND
Pigmentation	cream	red	pink	cream
Oxygen requirement	strictly aerobic	strictly aerobic	strictly aerobic	strictly aerobic
Gram strain	negative	negative	negative	negative
NaCl range (%,w/v)	15-30	15-30	10-30	11-31
NaCl optimum (%,w/v)	25	25	22.5-25	17-20
Temperature range (°C)	37-55	28-45	40-50	25-50
Temperature optimum (°C)	40	37	40	37-45
pH range	7 -11	6-11	6-9.2	6 -8
pH optimum	8	7.5-8	7.5	7.0
Motility	non-motile	non-motile	motile	non-motile
Catalase	+	+	+	+
				
**Hydrolysis of**				
Starch	-	-	+	-
Tween 80	+	+	+	-
Casein	+	-	-	ND
Gelatin	+	+	-	-
Lipids from egg yolk	+	ND	-	ND
				
**Utilization of**				
D-Glucose	+	+	+	+
Galactose		-	ND	-
D-Xylose	+	+	+	-
Lactose	-	-	-	-
Fructose	-	-	+	-
Starch	-	-	+	+
Mannose	+	-	ND	+
D-Ribose	+	-	ND	-
Sucrose	+	-	+	ND
Rhamnose	+	ND	ND	-
Mannitol	-	-	ND	ND
Citrate	+	-	ND	-
L-Arginine	-	-	-	-
Indole production	-	-	+	-
Urease	-	+	-	-
H2S production	-	-	+	+

Strains: *H. djelfamassiliensis* sp. nov. IIH2^T^; *H. xanaduensis* SH-6T; *H. aswanensis*; *H. salifodinae* KCY076B2^T^.

+: Positive result, -: Negative result, ND: Not Determined.

*Halopiger djelfamassiliensis* strain IIH2^T^ was susceptible to bacitracin (10 μg), novobiocin (30 μg) and tetracycline (30 μg) but resistant to ampicillin (10 μg), cephalothin (30 μg), chloramphenicol (30 μg), streptomycin (10 μg), erythromycin (15 μg), gentamicin (10 μg), kanamycin (30 μg), nalidixic acid (30 μg), penicillin G (10 μg) and vancomycin (30 μg).

**Figure 4:**
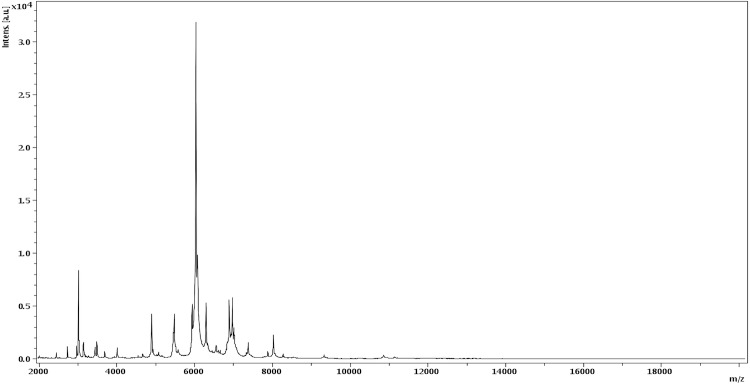
Reference mass spectrum from *H. djelfamassiliensis* strain IIH2^T^. Spectra from 12 individual colonies were compared and a reference spectrum was generated.

**Figure 5:**
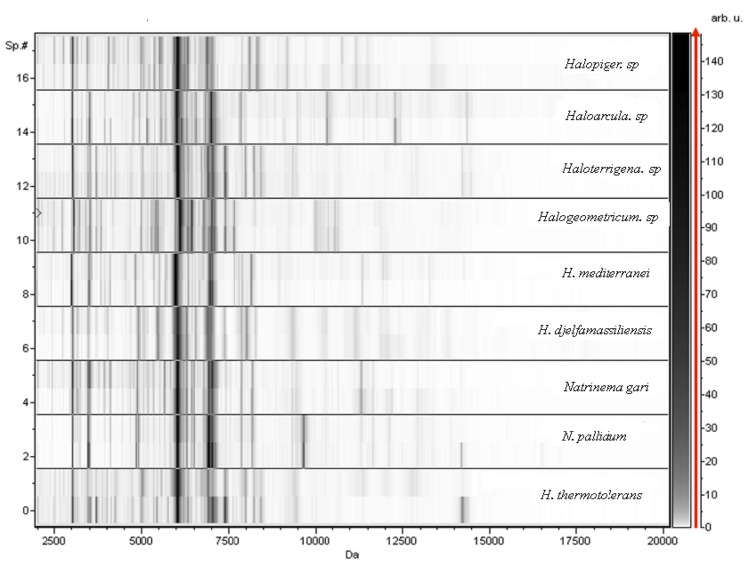
Gel view comparing the *H. djelfamassiliensis* strain IIH2^T^ spectrum with those of other archaea. The Gel View displays the raw spectra of all loaded spectrum files arranged in a pseudo-gel like look. The x-axis records the m/z value. The left y-axis displays the running spectrum number originating from subsequent spectra loading. The peak intensity is expressed by a Gray scale scheme code. The color bar and the right y-axis indicate the relation between the color a peak is displayed and the peak intensity in arbitrary units.

Matrix-assisted laser-desorption/ionization-time-of-flight (MALDI-TOF) mass spectrometry (MS) protein analysis was carried out as previously described [5,6] using a Microflex spectrometer (Bruker Daltonics, Germany). Briefly, a pipette tip was used to pick one isolated archaeal colony from a culture agar plate and spread it as a thin film on a MTP 384 MALDI-TOF target plate (Bruker Daltonics). Twelve distinct deposits were done for strain IIH2^T^ from 12 isolated colonies. Each smear was overlaid with 1.5 µL of matrix solution (a saturated solution of alpha-cyano-4-hydroxycinnamic acid) in 50% acetonitrile, 2.5% tri-fluoracetic acid and allowed to dry for 5 minutes. Spectra were recorded in the positive linear mode for the mass range from 2,000 to 20,000 Da. A spectrum was obtained after 675 shots with variable laser power. The time of acquisition was between 30 seconds and 1 minute per spot. The 12 IIH2^T^ spectra were imported into the MALDI Bio Typer software (version 2.0, Bruker) and analyzed by standard pattern matching (with default parameter settings) against the main spectra of 8 Archaea (*Natrinema gari*, *Natrinema pallidum*, *Haloterrigena thermotolerans*, *Haloterrigena. sp*, *Haloarcula. sp*, *Halopiger. sp, Haloferax mediterranei*, *Halogeometricum. sp*) used as reference data (Figures 4 and 5). The method of identification included the m/z from 2,000 to 20,000 Da. For every spectrum, 100 peaks at most were taken into account and compared with the spectra in the database. The MALDI-TOF score enabled the predictive identification and discrimination of the tested species from those in a database: a score > 2 with a validated species enabled identification at the species level, and a score < 1.7 did not enable any identification. No significant score was obtained for strain IIH2^T^ against the archaea database, suggesting that our isolate was not a member of a known species. We added the spectrum from strain IIH2^T^ to our database for future reference (Figure 4). Figure 5 shows the MALDI-TOF MS spectrum differences between *H. djelfamassiliensis* and other archaea (Figure 5).

## Genome sequencing information

### Genome project history

The organism was selected for sequencing on the basis of its phenotypic differences, phylogenetic position and 16S rRNA similarity to other members of the genus *Halopiger*, and as part of the study of archaeal diversity in hypersaline lakes of Algeria. It is the second genome of a *Halopiger* species and the first sequenced genome of *H. djelfamassiliensis* sp. nov. The EMBL accession number is CBMA010000001-CBMA010000055 and it consists of 6 scaffolds (HG315684-HG315689). Table 3 shows a summary of the project (PRJEB1777) information and its association with MIGS version 2.0 recommendations [24].

**Table 3:** Project information

**MIGS ID**	**Property**	**Term**
MIGS-31	Finishing quality	High-quality draft
MIGS-28	Libraries used	Paired-end 5 kb library
MIGS-29	Sequencing platforms	454 GS FLX Titanium
MIGS-31.2	Fold coverage	23.8x
MIGS-30	Assemblers	Newbler version 2.5.3
MIGS-32	Gene calling method	Prodigal
	EMBL ID	CBMA010000001-CBMA010000055
	EMBL Date of Release	June 18, 2013
	Project relevance	Study of the archaeal diversity in hypersaline lakes of Algeria

### Growth conditions and DNA isolation

*Halopiger djelfamassiliensis* strain IIH2^T^ sp. nov. (=CSUR P3035= DSM on-going deposit) was grown aerobically on SG medium at 40°C. Four petri dishes were spread and resuspended in 4×50µl of DTT buffer (60 mM). After incubation at 60°C for 20 min, proteinase K (0.2mg/mL) was added and the sample was incubated at 37°C for 2h. The lysate was extracted with an equal volume of buffered phenol followed by a classical phenol-chloroform extraction method [35]. The quality of the DNA was checked on an agarose gel (0.8%) stained with SYBR safe. The yield and the concentration were measured using the Quant-it Picogreen kit (Invitrogen) on the Genios_Tecan fluorometer at 126 ng/µl.

### Genome sequencing and assembly

A paired-end sequencing strategy was used (Roche). The library was pyrosequenced on a GS FLX Titanium sequencer (Roche). This project was loaded on a 1/4 region on PTP Picotiterplate (Roche). Three µg of DNA was mechanically fragmented on the Covaris device (KBioScience-LGC Genomics, Teddington, UK) using miniTUBE-Red 5Kb. The DNA fragmentation was visualized through the Agilent 2100 BioAnalyzer on a DNA labchip 7500 with an optimal size of 5.4 kb. After PCR amplification through 17 cycles followed by double size selection, the single stranded paired-end library was then loaded on a DNA labchip RNA pico 6000 on the BioAnalyzer. The pattern showed an optimal at 680 bp and the concentration was quantified on a Genios Tecan fluorometer at 456 pg/µL. The library concentration equivalence was calculated at 10^8^ molecules/µL. The library was stored at -20°C until further use. The library was clonally amplified in 2 emPCR reactions at 0.25, 0.5 and 1 cpb with the GS Titanium SV emPCR Kit (Lib-L) v2 (Roche). The yield of the 3 types of paired-end emPCR reactions was 4.09%, 5.69% and 11.31% respectively, in the quality range of 5 to 20% expected from the Roche procedure. These emPCR were pooled. Approximately 480,000 beads were loaded on the GS Titanium PicoTiterPlates PTP Kit 70x75 and sequenced with the GS FLX Titanium Sequencing Kit XLR70 (Roche). The run was performed overnight and then analyzed on the cluster through the gsRunBrowser and Newbler Assembler (Roche). A total of 264,150 filter-passed wells were obtained and generated 89.81 Mb of DNA sequences with a length average of 381 bp. The filter-passed sequences were assembled using Newbler with 90% identity and 40 bp overlap. The final assembly identified 54 large contigs (>1,500 bp) arranged into 6 scaffolds and generated a genome size of 3.77 Mb which corresponds to a coverage of 23.8× genome equivalent.

### Genome annotation

Open Reading Frames (ORFs) were predicted using prodigal [36] with default parameters. ORFs spanning a sequencing gap region were excluded. Assessment of protein function was obtained by comparing the predicted protein sequences with sequences in the GenBank [37] and the Clusters of Orthologous Groups (COG) databases using BLASTP. RNAmmer [38] and tRNAscan-SE 1.21 [39] were used for identifying the rRNAs and tRNAs, respectively. SignalP [40] and TMHMM [41] were used to predict signal peptides and transmembrane helices, respectively. ORFans of alignment length greater than 80 amino acids were identified if their BLASTP E-value was lower than 1e-03.. An E-value of 1e-05 was used if alignment lengths were smaller than 80 amino acids. DNA Plotter [42] was used for visualization of genomic features and Artemis [43] was used for data management. The mean level of nucleotide sequence similarity was estimated at the genome level between *H. djelfamassiliensis* and 5 other members of the *Halobacteriaceae* family (Table 6), by BLASTN comparison of orthologous ORFs in pairwise genomes. Orthologous proteins were detected using the Proteinortho software using the following parameters e-value 1e-05, 30% identity, 50% coverage and 50% of algebraic connectivity [44].

**Table 6:** Orthologous gene comparison and average nucleotide identity of *H. djelfamassiliensis* with other compared genomes (upper right, numbers of orthologous genes; lower left, mean nucleotide identities of orthologous genes). Bold numbers indicate the numbers of genes for each genome.

Species (accession number)	*H. djelfamassiliensis*	*N. pharaonis*	*H.* *turkmenica*	*N. magadii*	*H. jeotgali*	*H. xanaduensis*
*Halopiger djelfamassiliensis (*PRJEB1777)	**3761**	1405	2041	1889	1567	2197
*Natronomonas pharaonis* (NC_007426)	67.64	**2659**	1395	1330	1258	1383
*Haloterrigena turkmenica* (NC_013743)	79.24	67.76	**3739**	1771	1574	2031
*Natrialba magadii* (NC_013922)	77.19	66.81	76.97	**3559**	1443	1830
*Halalkalicoccus jeotgali* (NC_014297)	68.64	67.71	68.93	67.58	**3035**	1580
*Halopiger xanaduensis* (NC_015666)	79.38	67.52	79.88	77.00	68.91	**3588**

## Genomes properties

**Figure 6:**
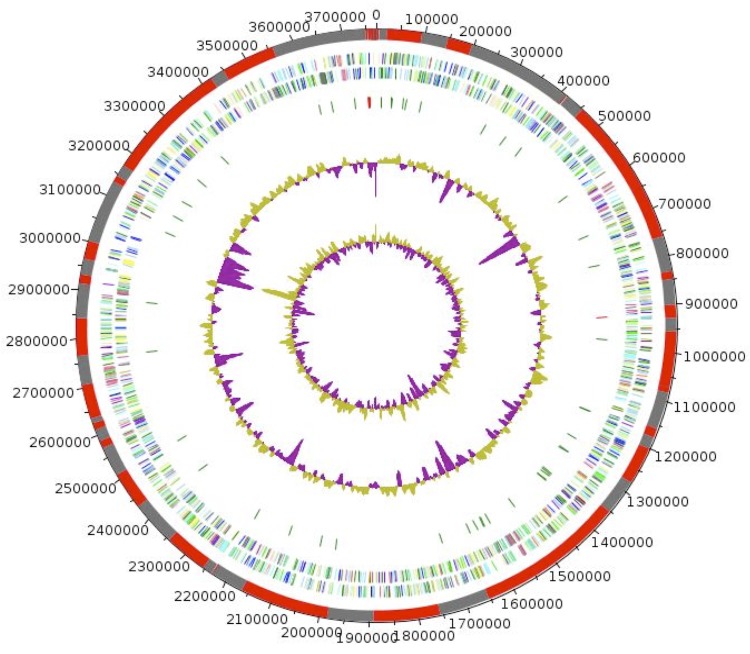
Graphical circular map of the *H. djelfamassiliensis* IIH2^T^ genome. From the outside in: the outer two circles show open reading frames oriented in the forward and reverse (colored by COG categories) directions, respectively. The third circle displays the rRNA gene operon (red) and tRNA genes (green). The fourth circle shows the G+C% content plot. The inner-most circle shows the GC skew, purple and olive indicating negative and positive values, respectively.

The genome is 3,771,216 bp long with 64,30% G+C content (Table 4, Figure 6). It is composed of 73 contigs (54 contigs are >1,500 bp) arranged into 6 scaffolds. Of the 3,812 predicted genes, 3,761 were protein-coding genes, and 51 were RNAs (1 gene is 16S rRNA, 1 gene is 23S rRNA, 2 genes are 5S rRNA, and 47 are tRNA genes). A total of 2,319 genes (61.66%) were assigned a putative function (by COG or by NR BLAST). In addition, 174 genes were identified as ORFans (4.63%). The remaining genes were annotated as hypothetical proteins (1035 genes = 27.52%). The distribution of genes into COG functional categories is presented in Table 4. The properties and the statistics of the genome are summarized in Tables 4 and 5.

**Table 4:** Nucleotide content and gene count levels of the genome

**Attribute**	**Value**	**% of total^a^**
Size (bp)	3,771,216	100
G+C content (bp)	2,424,851	64.30
Coding region (bp)	3,274,113	86.82
Total genes	3,812	100
RNA genes	51	1.34
Protein-coding genes	3,761	98.66
Genes with function prediction	2,319	61.66
Genes assigned to COGs	2,381	63.31
Genes with peptide signals	352	9.36
Genes with transmembrane helices	807	21.46

^a^ The total is based on either the size of the genome in base pairs or the total number of protein coding genes in the annotated genome.

**Table 5:** Number of genes associated with the 25 general COG functional categories

**Code**	**Value**	**%age**^a^	**Description**
J	165	4.39	Translation
A	1	0.03	RNA processing and modification
K	156	4.15	Transcription
L	123	3.27	Replication, recombination and repair
B	3	0.08	Chromatin structure and dynamics
D	20	0.53	Cell cycle control, mitosis and meiosis
Y	0	0	Nuclear structure
V	45	1.20	Defense mechanisms
T	96	2.55	Signal transduction mechanisms
M	84	1.23	Cell wall/membrane biogenesis
N	44	1.17	Cell motility
Z	0	0	Cytoskeleton
W	0	0	Extracellular structures
U	29	0.77	Intracellular trafficking and secretion
O	110	2.92	Post-translational modification, protein turnover, chaperones
C	184	4.89	Energy production and conversion
G	120	3.19	Carbohydrate transport and metabolism
E	252	6.70	Amino acid transport and metabolism
F	71	1.89	Nucleotide transport and metabolism
H	124	3.30	Coenzyme transport and metabolism
I	100	2.66	Lipid transport and metabolism
P	171	4.55	Inorganic ion transport and metabolism
Q	72	1.91	Secondary metabolites biosynthesis, transport and catabolism
R	484	12.87	General function prediction only
S	233	6.20	Function unknown
-	1380	36.69	Not in COGs

^a^ The total is based on the total number of protein coding genes in the annotated genome.

## Comparison with other genomes of *Archaea*

Currently, only one genome from *Halopiger* species is available. Here, we compared the genome of *H. djelfamassiliensis* strain IIH2^T^ with those of *H. xanaduensis* strain SH-6, *Halalkalicoccus jeotgali* strain B3, *Natronomonas pharaonis* strain DSM 2160, *Haloterrigena turkmenica* strain DSM 5511 and *Natrialba magadii* strain ATCC 43099. The draft genome of *H. djelfamassiliensis* (3.77 Mb) is larger than that of *Halalkalicoccus jeotgali* and *Natronomonas pharaonis* (3.69 and 2.75 Mb, respectively) but of a smaller size than *H. xanaduensis*, *Natrialba magadii* and *Haloterrigena turkmenica* (4.35, 4.44 and 5.44 Mb respectively). The G+C content (in %) of *H. djelfaamassiliensis* (64.30%) is higher than that of *Haloterrigena turkmenica* (64.26%), *Natronomonas pharaonis* (63.1%), *Halalkalicoccus jeotgali* (62.5%) and *Natrialba magadii* (61.1%) but smaller than *H. xanaduensis* (65.2%).

*H. djelfamassiliensis* has more predicted protein-coding genes (3,761) than *Haloterrigena turkmenica, H. xanaduensis, Natrialba magadii*, *Halalkalicoccus jeotgali* and *Natronomonas pharaonis* (3,739, 3588, 3,559, 3035 and 2,659 respectively). In addition, *H. djelfaamasiliensis* shared a mean genomic sequence similarity of 67.64, 79.24, 77.19, 68.64 and 79.38% with *Natronomonas pharaonis*, *Haloterrigena turkmenica*, *Natrialba magadii*, *Halalkalicoccus jeotgali* and *Halopiger xanaduensis* respectively (Table 6).

## Conclusion

On the basis of phenotypic, phylogenetic and genomic analyses, we formally propose the creation of *Halopiger djelfamassiliensis* sp. nov. that contains the strain IIH2^T^. This archaeal strain has been found in Algeria.

### Description of *Halopiger djelfamassiliensis* sp. nov.

***Halopiger djelfamassiliensis*** (dj. el. fa. ma. si. li. en’sis. L. gen. fem. n. djelfamassiliensis from the combination of **Djelfa,** the Algerian region where the strain was isolated, and massiliensis, of Massilia, the Latin name of Marseille, where the strain was sequenced). It has been isolated from an evaporitic sediment of the hypersaline Lake Zahrez Gharbi in the Djelfa region of Algeria.

Colonies were smooth, viscous and cream-pigmented with 3 to 4 mm in diameter on SG medium after incubation for 7 days at 40°C. Strain IIH2^T^ is a Gram-negative, non-motile, strictly aerobic and extremely halophilic archeon. Growth occurs at NaCl concentrations of 15-30%, at pH values in the range 7-11, and within the temperature range 37-55 °C. Optimal NaCl concentration, pH and temperature for growth are 25%, 8.0 and 40 °C, respectively. Magnesium is not required for growth. Cells are polymorphic (0.9-2.2 µm) and lyse in distilled water. Tween 80, gelatin and lipids from egg yolk are hydrolysed, D-glucose, D-melibiose, L-rhamnose, D-xylose, D-galactose, D-mannose, D-ribose and D-sucrose are fermented. Cells are susceptible to bacitracin, novobiocin and tetracycline but resistant to ampicillin, cephalothin, chloramphenicol, erythromycin, gentamicin, kanamycin, nalidixic acid, penicillin G, streptomycin, and vancomycin. The G+C content of the genome is 64.30%. The 16S rRNA and genome sequences are deposited in GenBank and EMBL under accession numbers KC430939 and CBMA010000001-CBMA010000055 respectively. The type strain IIH2^T^ (=CSUR P3035= DSM on-going deposit) was isolated from the sediment border of the hypersaline Lake Zahrez Gharbi, located in the Djelfa region of Algeria.
